# An Extract from *Ficus carica* Cell Cultures Works as an Anti-Stress Ingredient for the Skin

**DOI:** 10.3390/antiox10040515

**Published:** 2021-03-25

**Authors:** Irene Dini, Danila Falanga, Ritamaria Di Lorenzo, Annalisa Tito, Gennaro Carotenuto, Claudia Zappelli, Lucia Grumetto, Antonia Sacchi, Sonia Laneri, Fabio Apone

**Affiliations:** 1 Department of Pharmacy, University of Naples Federico II, Via Domenico Montesano 49, 80131 Napoli, Italy; dilorenzo@ritamaria.unina.it (R.D.L.); lucia.grumetto@unina.it (L.G.); antonia.sacchi@unina.it (A.S.); 2 Arterra Bioscience SpA, Via Benedetto Brin 69, 80142 Napoli, Italy; danila@arterrabio.it (D.F.); annalisa@arterrabio.it (A.T.); gennaro@arterrabio.it (G.C.); fapone@arterrabio.it (F.A.); 3 Vitalab Srl, Via Benedetto Brin 69, 80142 Napoli, Italy; claudiazappelli@vitalabactive.com

**Keywords:** *Ficus carica*, oxidative stress protection, lipid peroxidation, stress hormones, epidermal skin barrier, nutricosmetics

## Abstract

Psychological stress activates catecholamine production, determines oxidation processes, and alters the lipid barrier functions in the skin. Scientific evidence associated with the detoxifying effect of fruits and vegetables, the growing awareness of the long-term issues related to the use of chemical-filled cosmetics, the aging of the population, and the increase in living standards are the factors responsible for the growth of food-derived ingredients in the cosmetics market. A *Ficus carica* cell suspension culture extract (FcHEx) was tested in vitro (on keratinocytes cells) and in vivo to evaluate its ability to manage the stress-hormone-induced damage in skin. The FcHEx reduced the epinephrine (−43% and −24% at the concentrations of 0.002% and 0.006%, respectively), interleukin 6 (−38% and −36% at the concentrations of 0.002% and 0.006%, respectively), lipid peroxide (−25%), and protein carbonylation (−50%) productions; FcHEx also induced ceramide synthesis (+150%) and ameliorated the lipid barrier performance. The in vivo experiments confirmed the in vitro test results. Transepidermal water loss (TEWL; −12.2%), sebum flow (−46.6% after two weeks and −73.8% after four weeks; on the forehead −56.4% after two weeks and −80.1% after four weeks), and skin lightness (+1.9% after two weeks and +2.7% after four weeks) defined the extract’s effects on the skin barrier. The extract of the *Ficus carica* cell suspension cultures reduced the transepidermal water loss, the sebum production, the desquamation, and facial skin turning to a pale color from acute stress, suggesting its role as an ingredient to fight the signs of psychological stress in the skin.

## 1. Introduction

The focus on “cosmetics from food” has been a significant trend in recent years. The integration of natural ingredients from food into dietary supplements and cosmetic products is a reality that has already been commercialized. Fruits contain anti-aging, antioxidant, and anti-inflammatory molecules, and consequently, have a high potential in cosmetics [1,2,3]. The skin is the most exposed organ of our body such that its health is threatened by a large variety of environmental factors (temperature shifts, sun radiation, tobacco smoke, ozone, chemical pollutants), which is known as the “exposome.” The concept of the exposome includes the totality of factors to which an individual gets exposed from conception to death; this term entered our vocabulary quite recently [4]. Besides the exposome, additional factors have been recognized as skin-aging potentiators, acting separately or in combination with the exposome [5], such as psychological stress, lack of sleep, and inappropriate nutrition. Among these, psychological stress represents one of the most occurring issues nowadays. It can be defined as the condition in which environmental demands exceed the individual’s ability to cope, resulting in behavioral and mood changes [6]. Psychological stress determines the release of corticotropin releasting hormone (CRH), adrenocorticotropin (ACTH), glucocorticoids, catecholamines, epinephrine (adrenaline), and norepinephrine (noradrenaline) into the bloodstream in response to the unfavorable conditions [7]. If the stress response is persistent, those hormones may trigger adverse physiological events and exacerbate the stressful condition [8]. The skin and brain are strictly connected: the brain delivers signals to the skin that influences its stress response, mainly through the hypothalamic–pituitary–adrenal (HPA) axis, while the skin cells, in turn, produce a CRH, ACTH, epinephrine, and their receptors, triggering autocrine pathways and regulating the endocrine and immune systems [9]. Catecholamines stimulate keratinocyte differentiation by inducing keratin, involucrin, and transglutaminase [10], and exacerbate atopic dermatitis in an inflamed state via the continuous induction of a cytokine cascade [11]. Moreover, catecholamines, which are overproduced during psychological stress, impair the skin barrier’s recovery, reducing the accumulation of epidermal lipids—mainly cholesterol, fatty acids, and ceramides—by suppressing their synthetic pathways [12]. Chronic psychological stress strongly increases reactive oxygen species in mice skin, where these effects dramatically accelerate skin aging [13]. Oxidative stress in the skin is strictly related to the formation of age spots too. The accumulation of oxidized lipids and proteins culminates in the formation of lipofuscin particles, which are responsible, together with melanin, for dark spots [14] that the hydrolase enzymes hardly degrade. In this study, we evaluated the activity of an extract from *Ficus carica* cell suspension cultures to alleviate skin damage caused by psychological stress using in vitro and in vivo analyses. Plant cell cultures are becoming a popular source of cosmetic ingredients with proven efficacy [15], where their characteristics of safety and sustainability have been very much requested by the skincare market [16].

## 2. Materials and Methods

### 2.1. In Vitro Studies

#### 2.1.1. Plant Cell Culturing and Extract Preparation

A certified fig plant (*Ficus carica*) was obtained from a local nursery (“Vivai Milone,” Lamezia Terme, Calabria Region, Italy). The leaves were surface sterilized with 70% *(v/v)* EtOH (Sigma Aldrich, St. Louis, MO, USA), followed by a treatment with 1% bleach (supplemented with Tween 20 (Sigma Aldrich, St. Louis, MO, USA)), and finally rinsed with sterile distilled water. Then, the leaves were excised into 0.5–1.0 cm segments and cultured on solidified Gamborg B5 medium (Merk, Darmstadt, Germany) containing 500 mg/L myo-inositol, 30 g/L sucrose, phytohormones, and 8 g/L phytoagar. The explants were subcultured every four weeks onto a fresh medium for three months. Once a compact and friable callus was obtained, the cells were transferred to the liquid Gamborg B5 medium, supplemented with 500 mg/L myo-inositol, 30 g/L sucrose, 1 mg/L adenine, and phytohormones. The suspensions were shaken on a rotary shaker at 27 °C, with a 16-h daily photoperiod. Once the liquid suspension cultures of about 200 g/L were obtained, the cells were collected, lysed in a water-based buffer, and lyophilized to prepare a water-soluble extract. The powder (*Ficus carica* hydrosoluble extract (FcHEx)) was dissolved in water or cell culture media at appropriate testing concentrations.

#### 2.1.2. Skin Cells and Explants

Immortalized Human Keratinocytes (HaCaT), purchased from Addexbio Technologies (San Diego, CA, USA), were maintained in Dulbecco’s Modified Eagle Medium (DMEM; Sigma Aldrich, St. Louis, MO, USA) that was supplemented with 10% fetal bovine serum (FBS; Sigma Aldrich, St. Louis, MO, USA) in 95% air, 5% CO_2_, and humidified atmosphere at 37 °C.

Skin explants were obtained from the skin of healthy female donors (aged 35–49) following mastectomy or breast reduction procedures at the Villa Cinzia surgery center (Naples, IT). The use of skin tissue was done according to the Declaration of Helsinki and all patients had given their written informed consent. Skin punch biopsies (8 mm) were obtained from skin explants and cultured in 24-transwell plates in DMEM/FBS plus antibiotics in air-liquid conditions at 37 °C in 5% CO_2_ humidified air.

#### 2.1.3. Gene Expression Analysis

A total of 2 × 10^5^ HaCaT were seeded in six-well plates, incubated for 16 h, and then treated with the FcHEx or ICI-118,551 (Sigma Aldrich, St. Louis, MO, USA) for 4 h in the presence of 10 µM of epinephrine (Sigma Aldrich, St. Louis, MO, USA). At the end of the incubation, total RNA was extracted with the GenElute Mammalian Total RNA Purification Kit (Sigma Aldrich, St. Louis, MO, USA) and treated with DNAse I (Sigma Aldrich, St. Louis, MO, USA) at 37 °C for 30 min. Reverse transcription was performed using the RevertAid™ First Strand cDNA Synthesis Kit (Thermo Fisher Scientific, Dallas, TX, USA). RT-PCR was performed with the Quantum RNA™ kit (Thermo Fisher, Waltham, MA, USA) containing specific primers to amplify 18S rRNA, along with competimers that reduced the amplified 18S rRNA product within the range to be used as an endogenous standard. The amplification reactions were performed using the Mastercycler™ ProS (Eppendorf, Milan, Italy) with the following general scheme: 2 min at 94 °C, followed by 35 cycles of 94 °C for 30 s, 50 °C for 30 s, and 72 °C for 30 s, with a 10 min final extension at 72 °C. The PCR products were loaded onto 1.5% agarose gel (Merk, Darmstadt, Germany), and the amplification bands were visualized and quantified with the Geliance 200 Imaging system (Perkin Elmer, Waltham, MA, USA). The amplification band corresponding to the analyzed gene was normalized relative to the amplification band corresponding to the 18S and reported as a percentage of untreated controls (fixed at 100%). The RT-PCRs were done in triplicate, where the average results are reported in the graphs. The sequences of the used primers were as follows: interleukin 6 (IL-6) forward (Fw)—GTCCTGATCCAGTTCCTGCAG, IL-6 reverse (Rv)—CTACACTTTCCAAGAAATGATC (gene ID: 3569), β-glucocerebrosidase (GBA) Fw—AGTTGCACAACTTCAGC, and GBA Rv—GTCCAGGTACCAATGTAC (gene ID: 2629).

#### 2.1.4. Determination of the Lipid Peroxides

A total of 1.8 × 10^4^ HaCaT were seeded in 96-well plates and then treated with the FcHEx or ICI-118,551 for 24 h. After incubation, the cells were washed in phosphate-buffered saline (PBS; Sigma Aldrich, St. Louis, MO, USA) and then incubated with the dye C11-Bodipy (Thermo Fisher, Waltham, MA, USA) at 37 °C for 30 min. A total of 50 µM of epinephrine was used to induce lipid peroxidation, and the fluorescence was read at 510/580 using the Victor3 plate reader system (Perkin Elmer, Waltham, MA, USA).

#### 2.1.5. Lipofuscin Measurements

A total of 8 × 10^3^ HaCaT were seeded in a 96-well plate and then treated with the FcHEx for 24 h. A total of 50 nM of epinephrine was added to the cells and incubated for an additional 48 h. The cells were then washed in PBS and incubated with Sudan Black (Sigma Aldrich, St. Louis, MO, USA) (0.7% dilution in EtOH 70%) for 5 min. The cells were lysed in 1 M of NaOH (Sigma Aldrich, St. Louis, MO, USA) for 20 min at 70 °C to solubilize the dye. The quantity of solubilized dye was determined by reading the absorbance at 595 nm in the Victor3 plate reader system.

#### 2.1.6. Epidermal Lipid Measurements

A total of 1.0 × 10^4^ HaCaT were seeded in 96-well plates, grown for 48 h, and then treated with the FcHEx or ICI-118,551 as a positive control for an additional 24 h. The cells were incubated with 10 µM of epinephrine for 24 h, and the lipid accumulation was detected via the addition of the AdipoRed assay Reagent (Lonza, Basel, Switzerland). The fluorescence at 485 nm was read using the Victor3 plate reader system, and the content of the epidermal lipid was normalized relative to the cell density determined by crystal violet staining.

#### 2.1.7. Measuring the Carbonylated Proteins in Cells and Skin Explants

A total of 1.5 × 10^4^ HaCaT were seeded in 96-well plates and then incubated with the FcHEx or ICI-118,551 in the presence of epinephrine 50 µM for 24 h. The cells were then washed in PBS and fixed in 4% paraformaldehyde (PFA; Sigma Aldrich, St. Louis, MO, USA). After washing in PBS + 0.05% Tween 20, the samples were incubated with 5 mM 2,4-Dinitrophenyl-hydrazine (DNPH; Sigma Aldrich, St. Louis, MO, USA) in 2N HCl (Sigma Aldrich, St. Louis, MO, USA) for 1 h at room temperature. The carbonylated products were detected via ELISA using a specific antibody against DNP (sc69697, Santa Cruz Biotechnology, Heidelberg, Germany).

The skin punches were treated with the FcHEx in the presence of 56 nM of epinephrine for 24 h. After incubation, the punches were fixed with PFA for 6 h, washed in PBS, and then incubated in 15% and 30% sucrose (Sigma Aldrich, St. Louis, MO, USA). The punches were embedded in optimal cutting temperature (OCT) medium (Tissue Tek, Sakura Finetek USA, Inc., Torrance, CA, USA), frozen in dry ice, and stored −80 °C. Cryosections (10 µm) were obtained using the CM1520 cryostat (Leica Microsystems, Wetzlar, Germany). The slides were incubated with 5 mM DNPH in 2N HCl for one hour at room temperature, washed in PBS/EtOH (1:1), and then in PBS/Tween 20. The slides were incubated in a blocking buffer (3% bovine serum albumin (BSA) in PBS) for 30 min, washed with PBS/Tween 20, incubated with the primary antibody anti-DNP (1:50 dilution), and then with the secondary conjugated antibody Alexa Fluor 488 (Life Technologies, Carlsbad, CA, USA). Nuclei of the cells were stained using 4′,6-Diamidino-2-phenylindole (DAPI; Sigma Aldrich, St. Louis, MO, USA). The signals were detected using fluorescent microscopy.

#### 2.1.8. cAMP Measurements

The HaCaT cells were washed in PBS, detached from the flask with a non-enzymatic dissociation solution (Sigma-Aldrich, St. Louis, MO, USA), and resuspended at the concentration of 10^6^/mL in a stimulation buffer (BSA 0.1%, isobutylmethylxanthine (IBMX) 0.5% in PBS) containing Alexa Fluor 647-labeled anti-cAMP antibody (Thermo Fisher Scientific, Dallas, TX, USA), according to the protocol described by the provider of the Lance Kit (Perkin Elmer, Waltham, MA, USA). A total of 1.2 × 10^4^ cells were distributed in aliquots in 384-well plates and treated with either the FcHEx or ICI-118,551 in the presence of 10 µM of epinephrine. In parallel, a standard curve for cAMP (Thermo Fisher Scientific, Dallas, TX, USA) was prepared by diluting known concentrations of cAMP in the stimulation buffer in the presence of an anti-cAMP antibody. The cells were incubated for 1 h at room temperature, and then a detection mix containing Europium-W8044-labeled streptavidin and biotin-labeled cAMP (Thermo Fisher Scientific, Dallas, TX, USA) was added to each well. The cAMP concentration was measured by exciting at 320 nm and recording at 615 and 665 nm using an EnVision instrument (Perkin Elmer, Waltham, MA, USA).

#### 2.1.9. Determination of the Ficin Amount and Activity

The ficin amount was determined using ELISA: scalar dilutions of the extract were incubated on a 96-well plate at 4 °C for 16 h, and then the quantity of ficin was calculated using a specific antibody (AS09550, Agrisera, Vännäs, Sweden). Scalar dilutions of the extract were incubated with 0.43 mM of the substrate pGLU-PHE-LEU-pNitroanilide (Sigma Aldrich, St. Louis, MO, USA) in a pH 6.5 phosphate buffer (Sigma Aldrich, St. Louis, MO, USA) to measure the ficin activity. Purified ficin was used as standard starting from 0.015 enzymatic units. The color developed was measured by reading the absorbance at 405nm with the Victor3 multiplate reader system.

#### 2.1.10. Statistical Analysis

The in vitro experiments were performed in triplicates and repeated at least three times. The data are expressed as mean ± standard deviation (SD) of three independent experiments. The statistical tests were performed with the aid of the GraphPad Prism version 9 software (GraphPad Software, San Diego, CA, USA). A two-way analysis of variance (ANOVA) was used, followed by a Tukey’s multiple comparisons post-test; a *p*-value lower than 0.05 was considered statistically significant. The number of asterisks in the graphs indicate the level of significance (*** *p*-value < 0.001; ** 0.001 < *p* < 0.01; * 0.01 < *p* < 0.05).

### 2.2. In Vivo Studies

The studies were carried out according to the principles of the 1964 Helsinki Declaration European Community revisions (fourth revision, called Somerset West, South Africa [17], and the May 2008 Colipa Guidelines for the estimation of a cosmetic product’s efficiency [18]. The study was conducted on forty healthy volunteers, aged between 20 and 27 years, going through psychological stress. They were preparing for a 15-credit exam. All the volunteer students successfully passed the exam (scores of 27/30–30/30). The measures were performed two weeks before the examination (*T_0_*), the day of the exam (acute stress time—*T_2w_*), and a successive period of two weeks (recovery time—*T_4w_*). These subjects provided informed consent and received an objective evaluation using standardized numerical scales, i.e., they were subjected to a Complete Skin Investigation (CSI; Courage + Khazaka Electronic GmbH, Cologne, Germany version for windows ^®^10) analysis for the evaluation of the degree of skin and the type of skin. In this study, twenty volunteers used an active formulation and twenty used a placebo in a double-blind trial.

Each volunteer was examined in a closed room, which was kept at a controlled temperature and humidity (20 ± 2 °C, 40 ± 5% relative humidity (RH)) at the same time of day after a stationing time of about 30 minutes.

The probes were previously calibrated in the same room, and the measurements were carried out at pre-established times (*T_0_*, *T_2w_*, *T_4w_*). The panelists applied about 2 mg/cm^2^ of cream on the face and eye area, which had been previously cleaned, avoiding direct contact with the eyes and gently massaging until completely absorbed.

At the beginning (*T_0_*), after 2 weeks (*T_2w_*), and at the end of the study period (after 4 weeks, *T_4w_*), the following instrumental evaluations were carried out:

Tewameter^®^ TM300 (Courage + Khazaka Electronic GmbH, Mathias-Brüggen-Str. 91 50829, Cologne, Germany) measured the transepidermal water loss.

Sebufix^®^ F 16 and Corneofix^®^ F 20 applied to a Visioscope PC35^®^ camera (Courage + Khazaka Electronic GmbH, Cologne, Germany) measured the sebum secretion. The Sebufix^®^ F 16 is a special foil that absorbs the skin surface’s sebum due to its micropores and shows them as spots of different sizes. The Visioscope PC35^®^ camera monitored the qualitative sebum production in real-time. The number, size, and area covered with spots (mm^2^) were evaluated for five increasing spot sizes, together with the slope of the sebum development during the measurement time on the cheeks and foreheads.

Corneofix^®^ F 20 (Courage + Khazaka Electronic GmbH, Cologne, Germany) special adhesive tapes collected flakes of dead cells, known as corneocytes. The number, size, and thickness of the corneocytes is correlated with the exfoliation of the stratum corneum. When mounted on the Visioscope PC35^®^ camera, the exfoliation can be evaluated using its software.

Visioface 1000D (Courage + Khazaka Electronic GmbH, Cologne, Germany) for images confirmed the pore counting and type.

The skin lightness evaluation was undertaken using a Colorimeter CL 400 (Courage + Khazaka Electronic GmbH, Cologne, Germany). The Colorimeter CL 400 probe sends out white LED light arranged circularly to illuminate the skin uniformly. The emitted light is scattered in all directions. Parts of it travel through the layers and some of it scatters out of the skin. The light reflected from the skin is measured in the probe and expressed accordingly. The probe’s raw data are corrected with a particular color matrix to be as close as possible to norm values. The skin’s color is expressed as an XYZ-value (tristimulus) and can be converted into RGB values (red/green/blue) or L*a*b.

A Skin-pH-Meter PH 905 (Courage + Khazaka Electronic GmbH, Mathias-Brüggen-Str. 91 50829, Cologne, Germany) measured the cute pH. The measurements were based on a high-quality combined electrode, where both a glass H^+^-ion-sensitive electrode and a reference electrode were placed in one housing. It was connected to a probe handle containing the measurement electronics.

The data analyses were obtained using CK-MPA-Multi-Probe-Adapter FBQ software version for windows ^®^10 (Courage + Khazaka Electronic GmbH, Cologne, Germany).

#### 2.2.1. Study Population

Forty healthy volunteers, female (*n* = 38) and male (*n* = 2), from all social categories, were enrolled according to these inclusion criteria:

- Healthy Caucasian subjects.
- Aged between 20 and 27 years and going through a period of psychological stress two weeks before the examination *T_0_*, the day of the examination (*T_2w_*—stress time), and 2 weeks after the examination (*T_4w_*—recovery time).
- Sex: female and male.
- Phototype: II–IV Fitzpatrick scale.
- Subjects who have read and signed the informed consent form written by the investigators.
- Subjects with stressed skin, as assessed via CSI analysis.
- Subjects who did not apply products other than the one studied on the test area and no product within seven days before the test.
- Subjects who agreed to follow the study rules and the planned check-ups.
- Subjects who agreed not to expose themselves to UV for the duration of the study.
- Exclusion criteria:
- Pregnant or breastfeeding women.
- Subjects with anamnesis of cutaneous hyper-reactivity or intolerance reactions to cosmetic products/ingredients.
- Subjects with diseases in the period immediately preceding the current study.
- Subjects undergoing topical or systemic treatment with any drug that may affect the outcome of the test or subjects affected by skin diseases (eczema, psoriasis, lesions).
- Subjects treated with topical retinoids in the previous six months at the start of the study or with systemic retinoids in the previous 12 months.
- Subjects who performed treatments with topical products based on alpha and beta-hydroxy acids in the 45 days before the start of the study.

#### 2.2.2. Cream Composition

The cream with *Ficus carica* cell suspension culture extract (FcHEx) contained two phases:

- Phase O (oil phase) polyglyceryl-3 methylglucose 5.0%, cetyl alcohol 2%, and cetearyl alcohol 3%.
- Phase W (water phase) containing water (88.8%), the FcHEx (0.5%), sodium benzoate 0.5%, potassium sorbate, and perfume (0.1%).

The placebo cream contained all the components without the FcHEx.

All components that were used to produce the creams were bought from ACEF (Fiorenzuola D’Arda, Italy) and Parfum by Farotti Essenze (Rimini, Italy). The two creams were produced by energetically shaking the two phases at 70 °C using a Silverson L5M-A Laboratory Mixer (SBL, Shanghai, China), cooled in an ice bath, and adding the remaining components at room temperature. Finally, the pH (5.1–5.2) and viscosity (28.179–29.287 mPa; L4, 20 rpm) were tested using a Crison GPL20 pH-Meter (Crison, Barcelona, Spain) and a Visco Basic Plus rheometer (Fungilab, Barcelona, Spain).

#### 2.2.3. Data Analysis and Statistics

The measurement averages were considered at the times *T_0_*, *T_2w_*, and *T_4w_*. Student’s *t*-test was used to assess whether the differences between the averages were significant, where the significance level was set to *p* ≤ 0.05. The mean value and standard deviation were calculated for the initial, intermediate, and final instrumental values using a spreadsheet by setting:

*T_0_* = average value before the study started;
*T_2w_* = average value after 2 weeks of treatment;
*T_4w_* = average value after 4 weeks of treatment;
*T_2w_* − *T_0_* = average value variation after 2 weeks;
*T_4w_* − *T_0_* = average value variation after 4 weeks.

This difference is also reported as a relative percentage of variation (∆%).

## 3. Results

### 3.1. In Vitro Studies

#### 3.1.1. Extract Preparation

A hydrosoluble extract was obtained from the *Ficus carica* liquid suspension culture by disrupting the cells in a saline buffer and taking the water-soluble supernatant (FcHEx). *Ficus carica* cell cultures are rich in polyphenols (flavones, flavanones, catechins, anthocyanidins, and phenolic acids) [19]. The concentration of ficin, a sulfhydryl protease with a papain-like activity that is abundant in the fig latex [20], was measured in the FcHEx using a specific antibody in ELISA, as its presence was already reported in *F. carica* cell cultures [21]. The concentration of ficin was about 1.48 µg/mg of dried extract, which was higher than that previously reported. The ficin activity was measured using an enzymatic assay and was found to be 0.9 enzymatic units/µg of extract. The extract concentrations to be used in the cellular tests were calculated via the MTT (3-(4,5-dimethylthiazol-2-yl)-2,5-diphenyltetrazolium bromide) cytotoxicity assay on the keratinocyte cell lines (HaCaT), treated with increasing concentrations of the FcHEx (from 0.01 to 0.0002% *w/v*). No significant cytotoxicity was recorded at all for the used concentrations (data not shown). For convenience, the two doses of 0.006% and 0.002% of the extract were chosen for all the following experiments.

#### 3.1.2. Activity of the FcHEx on Epinephrine Signaling

**Figure 1:**
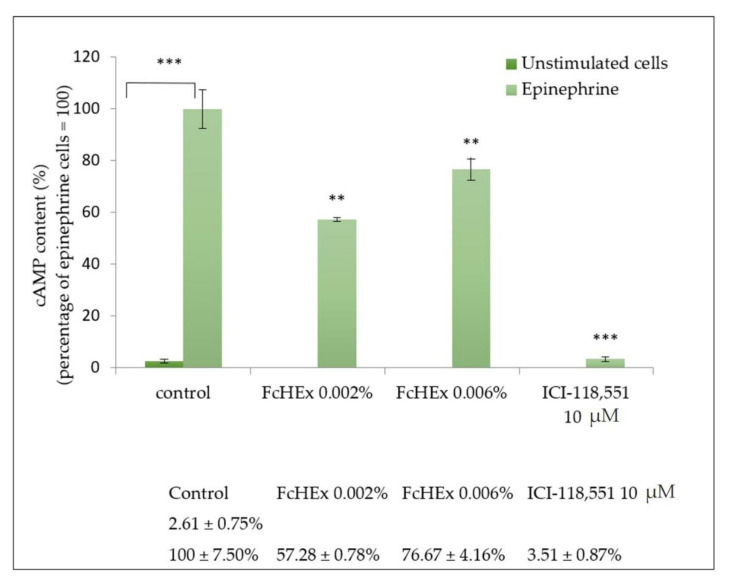
Effect of the *Ficus carica* cell suspension culture extract (FcHEx) on the cAMP synthesis in skin keratinocytes. The keratinocytes were stimulated with 10 µM of epinephrine alone or with the FcHEx or with a positive control for 30 min and then processed to determine the cAMP content. The reported values represent the averages of three independent experiments and are expressed as the percentages of cells treated with epinephrine only, which was set to 100%. The asterisks indicate statistically significant values (*** *p*-value was between 0.0001 and 0.001; ** *p*-value was between 0.001 and 0.01).

Keratinocytes were stimulated with epinephrine alone or in the presence of the FcHEx at the two concentrations, and the level of the second messenger cAMP was measured to investigate the capacity of the FcHEx to affect the epinephrine signal cascade in the skin cells. The ICI-118,551 was used as a positive control, as it inhibits the β2 adrenergic receptors [22]. As shown in Figure 1, the 10 µM of epinephrine stimulated the cAMP synthesis by about 40-fold, but this increase was attenuated by the FcHEx (about 43% and 24% at the concentrations of 0.002% and 0.006%, respectively). As expected, at a concentration of 10 µM, the ICI-118,551 totally abolished the epinephrine stimulation.

#### 3.1.3. Activity of the FcHEx on the Inflammatory Cytokine IL-6

**Figure 2:**
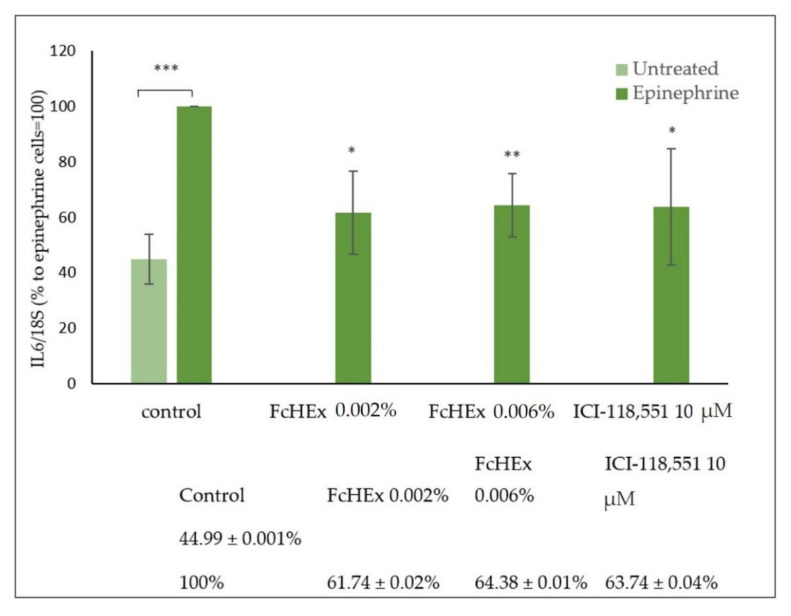
Effect of the FcHEx on the IL-6 gene expression in the skin keratinocytes. The keratinocytes were stimulated with 10 µM of epinephrine alone or with the FcHEx or the positive control for 4 h and then processed for RT-PCR analysis. The reported values represent the averages of three independent experiments and are expressed as the percentages of cells treated with epinephrine only, which was set to 100%. The asterisks indicate statistically significant values (*** *p*-value was between 0.0001 and 0.001; ** *p*-value wa between 0.001 and 0.01; * *p*-value was between 0.01 and 0.05).

The IL-6 gene expression was measured in keratinocytes treated with the FcHEx after stimulation with 10 µM of epinephrine, as it activates the synthesis of inflammatory cytokines through cAMP [23]. As shown in Figure 2, the IL-6 gene expression increased by about 50% in stimulated cells, but this effect was significantly reduced by the extract (38.3% and 35.6% at the concentrations of 0.002% and 0.006%, respectively) and the positive control ICI-118,551.

#### 3.1.4. Lipid Peroxide Measurements

**Figure 3:**
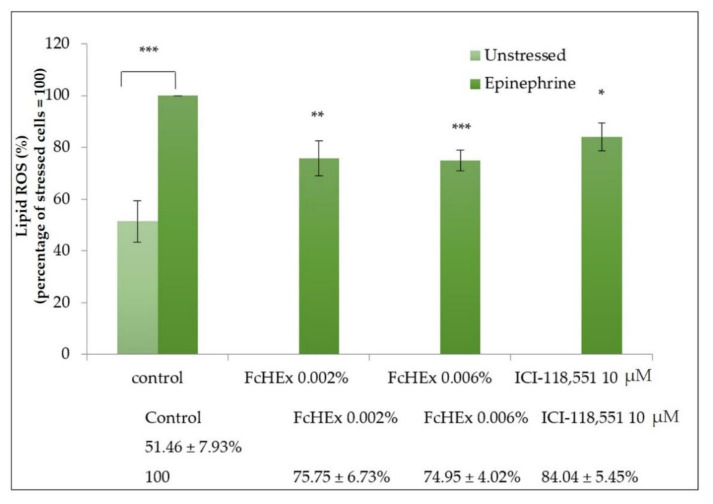
Effect of the FcHEx on the lipid peroxidation in skin keratinocytes. The keratinocytes were treated with the FcHEX or the positive control for 24 h, stressed with 50 µM of epinephrine, and then analyzed for lipid peroxide content by using C11-Bodipy dye. The reported values represent the average of three independent experiments and are expressed as the percentages of cells treated with epinephrine only, which was set to 100%. The asterisks indicate statistically significant values (*** *p*-value was between 0.0001 and 0.001; ** *p*-value was between 0.001 and 0.01; * *p*-value was between 0.01 and 0.05).

The FcHEx’s ability to reduce the oxidative stress induced by epinephrine was investigated by measuring the lipid peroxide production in keratinocytes. As shown in Figure 3, 50 µM of epinephrine increased the lipid peroxide production by 50%. Pretreatment with the FcHEx (at both the used concentrations) decreased this event by 25%.

#### 3.1.5. Carbonylated Protein Determination

**Figure 4:**
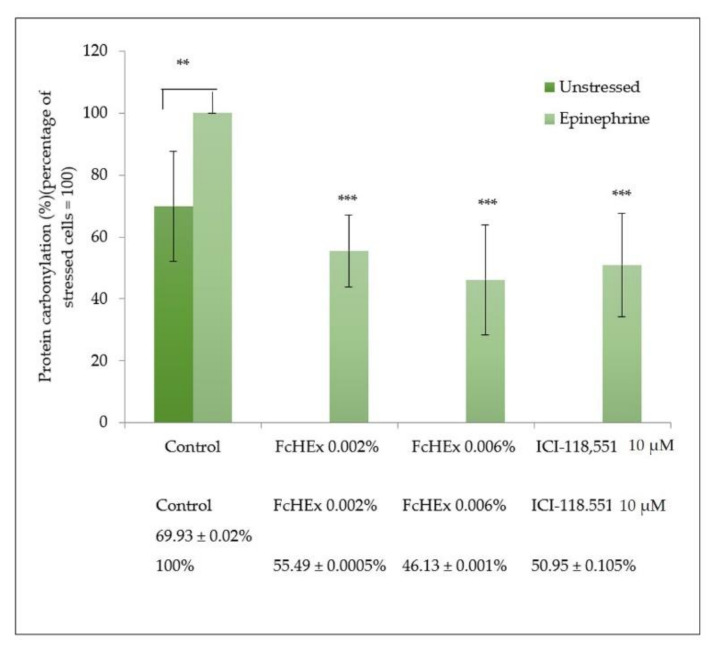
Effect of the FcHEx on the protein carbonylation in the skin keratinocytes. The keratinocytes were treated with the FcHEX or the positive control in the presence of 50 µM epinephrine for 24 h, fixed with formaldehyde, stained with DNPH (2,4-dinitrophenylhydrazine), and incubated with the specific antibody. The reported values represent the averages of three independent experiments and are expressed as the percentages of cells treated with epinephrine only, which was set to 100%. The asterisks indicate statistically significant values (*** *p*-value was between 0.0001 and 0.001; ** *p*-value was between 0.001 and 0.01).

The concentration of carbonylated proteins was measured in the keratinocytes stressed with epinephrine 50 µM and then treated with the FcHEx using a specific antibody to verify whether the lipid peroxide inhibition was associated with a reduction in protein oxidation. Epinephrine induced an increase in protein carbonylation by 40%, which was significantly inhibited by the extract (about 50%) and the inhibitor ICI-118,551 (Figure 4).

**Figure 5:**
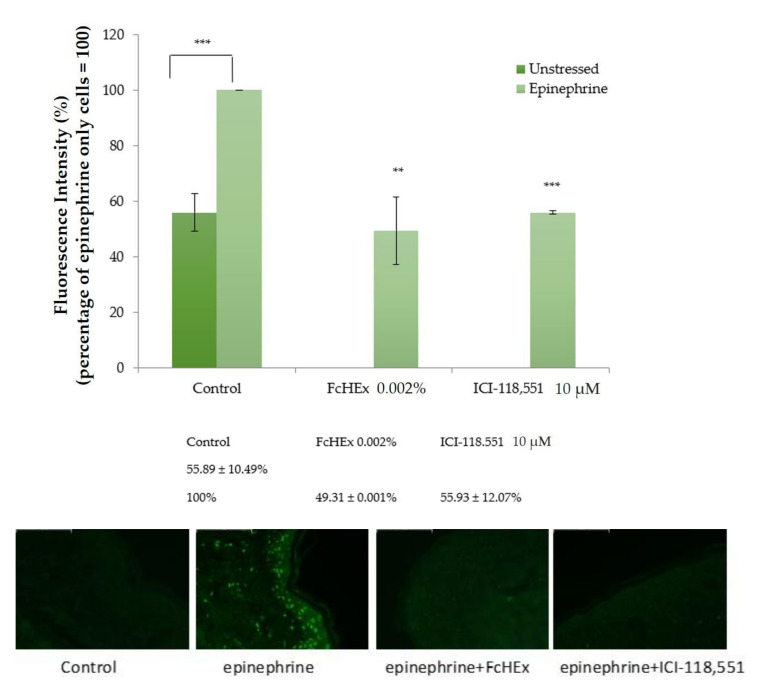
Effect of the FcHEx on the protein carbonylation in the human skin explants. Human skin punches, which were derived from aesthetic surgical biopsies, were treated with the FcHEx or the positive control in the presence of 56 nM of epinephrine. The reported values represent the averages of three independent experiments and are expressed as the percentages of skin explants treated with epinephrine only, which was set to 100%. The asterisks indicate statistically significant values (*** *p*-value was between 0.0001 and 0.001; ** *p*-value was between 0.001 and 0.01).

The protection against protein carbonylation of the FcHEx was evaluated in the skin explants as well. Three punches from biopsy explants were topically treated for each condition. The induction of protein carbonylation was achieved with 56 nM of epinephrine, which corresponded to the hormone dose found in psychologically stressed individuals’ blood plasma [24]. After the treatment with epinephrine, the level of carbonylated proteins increased by about 45%, while the treatment with the FcHEx 0.002% reduced this effect by 50%, analogously to the inhibitor drug (Figure 5).

#### 3.1.6. Lipofuscin Measurements

**Figure 6:**
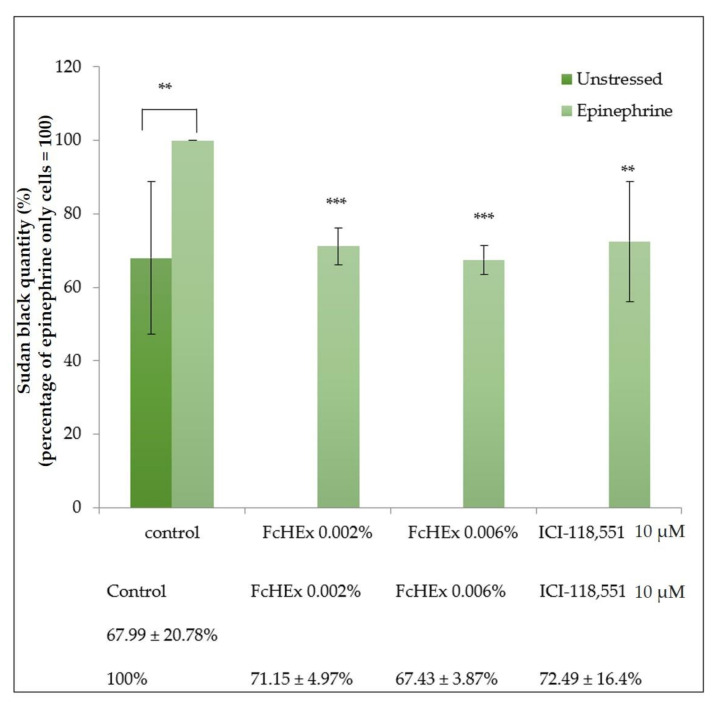
Effect of the FcHEx on the lipofuscin accumulation in the skin keratinocytes. The keratinocytes were treated with the FcHEx or the positive control for 24 h, then 50 nM of epinephrine was added to the cells incubated for an additional 48 h. At the end of the incubation, the cells were stained and the absorbance at 595 nM was measured. The obtained values were normalized relative to the protein content determined using a Bradford assay. The reported values represent the averages of three independent experiments and are expressed as the percentages of cells treated with epinephrine only, which was set to 100%. The asterisks indicate statistically significant values (*** *p*-value was between 0.0001 and 0.001; ** *p*-value was between 0.001 and 0.01).

The cells were stressed with epinephrine in the absence and presence of the extract, and the concentration of lipofuscin particles was measured as the accumulation of oxidated compounds, either lipids or proteins, which can often determine lipofuscin production. The treatment with epinephrine significantly increased the lipofuscin accumulation; in contrast, the treatment with both concentrations of the FcHEx decreased significantly (Figure 6).

#### 3.1.7. Activity of the FcHEx on the Ceramide Production

**Figure 7:**
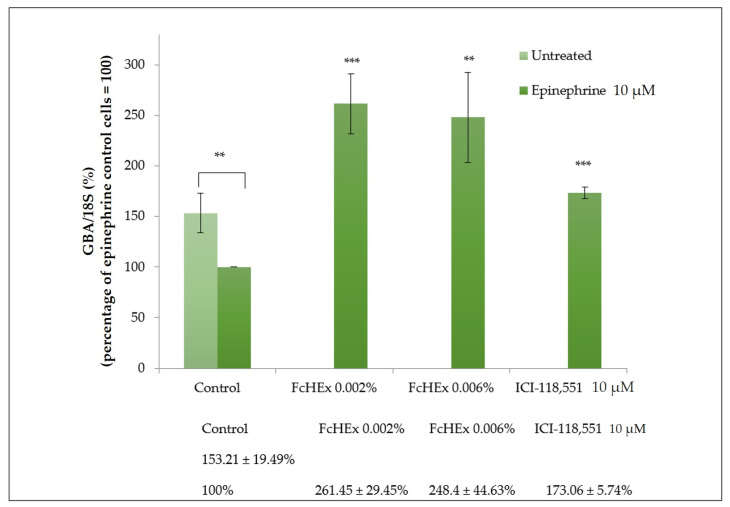
Effect of the FcHEx on the β-glucocerebrosidase (GBA) enzyme gene expression in the skin keratinocytes. The keratinocytes were stimulated with 10 µM of epinephrine alone or with the FcHEx or the positive control for 4 h and then processed for RT-PCR analysis. The reported values represent the averages of three independent experiments and are expressed as the percentages of cells treated with epinephrine only, which was set to 100%. The asterisks indicate statistically significant values (*** *p*-value was between 0.0001 to 0.001; ** *p*-value was between 0.001 to 0.01).

The gene expression of the GBA enzyme, which is responsible for the hydrolysis of glucocerebrosides into ceramides, and the amount of newly synthesized ceramides were measured to investigate the FcHEx’s ability to preserve the epidermal skin barrier against epinephrine stress. The treatment with the FcHEx increased the GBA expression by almost 150% in cells stressed with epinephrine (Figure 7).

**Figure 8:**
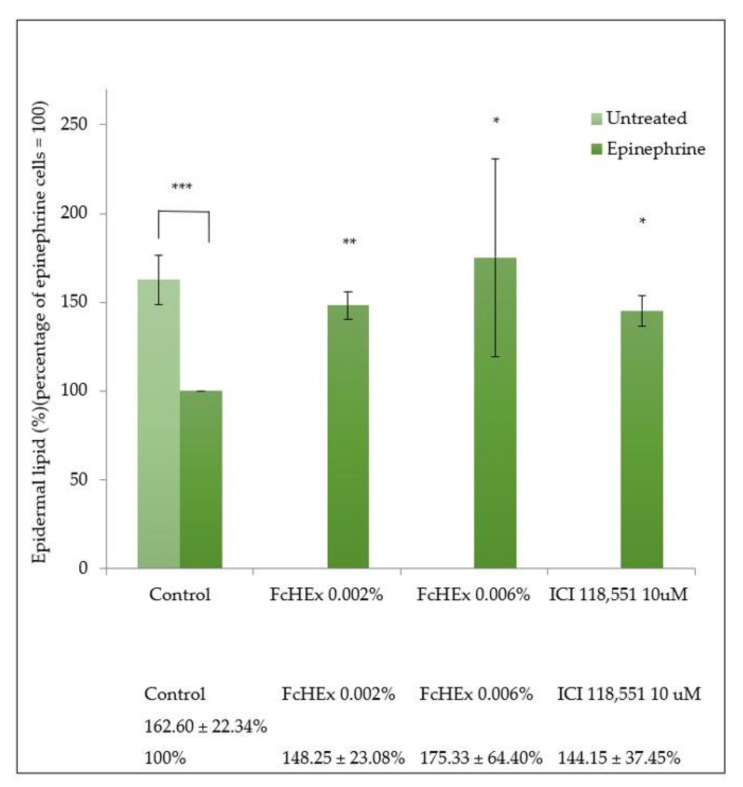
Effect of the FcHEx on the epidermal lipid content in the skin keratinocytes. The HaCaT cells were treated with the FcHEx or the positive control for 24 h, then incubated with epinephrine 10 µM for an additional 24 h. The lipid content was measured using an Adipored assay. The values were normalized relative to the cell density determined using crystal violet staining. The reported values represent the averages of three independent experiments and are expressed as the percentages of cells treated with epinephrine only, which was set to 100%. The asterisks indicate statistically significant values (*** *p*-value was between 0.0001 and 0.001; ** *p*-value was between 0.001 and 0.01; * *p*-value was between 0.01 and 0.05).

The concentration of ceramides was measured using Nile red staining in cells stressed with epinephrine and treated with the FcHEx. The ceramide content decreased in the epinephrine-treated keratocytes. In contrast, it significantly increased when the FcHEx was administered at both the used concentrations (Figure 8).

### 3.2. In Vivo Studies

#### 3.2.1. Transepidermal Water Loss (TEWL) Evaluation

**Figure 9:**
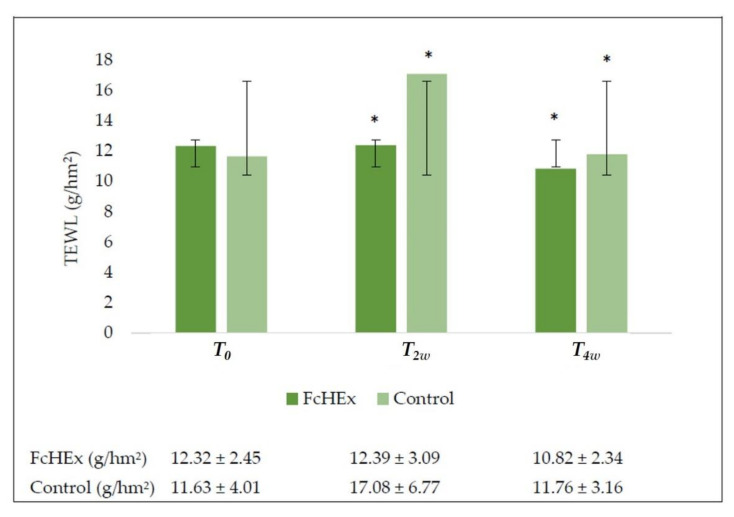
Transepidermal water loss evaluation (TEWL) evaluation. The TEWL trend values at time *T_0_* (before the application), *T_2w_* (after two weeks—acute stress), and *T_4W_* (after four weeks—recovery effect). All the measurements for *T_2w_* vs. *T_0_* and *T_4w_* vs. *T_0_* for the FcHEx and the control were statistically significant. The asterisks indicate statistically significant values (* *p*-value was between 0.01 and 0.05).

Stress is known to affect skin barrier permeability and negatively alter TEWL values. An evaporimeter evaluated the TEWL values were expressed in grams per square hectometer. As shown in Figure 9, the cream containing the extract counteracted the TEWL increase. During acute stress, i.e., after two weeks, a peak in the TEWL values in volunteers applying the placebo was observed, while the extract abolished this increase. After four weeks, the extract reduced the TEWL values by 12.2% during the recovery time, showing barrier recovery efficacy, while the placebo showed no significant effect.

#### 3.2.2. Sebum Production Determination

**Figure 10:**
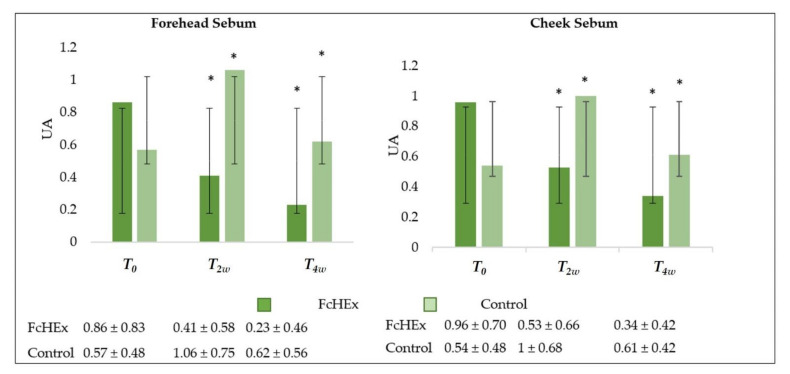
Sebum production evaluation on the forehead and cheek of volunteers. The sebum production trend values at time *T_0_* (before the application), *T_2w_* (after two weeks—acute stress), *T_4w_* (after four weeks—recovery effect). All the measurements for *T_2w_* vs. *T_0_* and *T_4w_* vs. *T_0_* for the FcHEx and the control were statistically significant. The asterisks indicate statistically significant values (* *p*-value was between 0.01 and 0.05).

The sebum secretion was monitored using a Sebufix^®^ F 16 and a Corneofix^®^ F 20 applied to a Visioscope PC35^®^ camera. After two and four weeks of use, a statistically significant decrease in the sebum concentration on the cheek and forehead in the volunteers treated with cream containing the FcHEx was recorded. More precisely, sebum production on the cheek decreased by 46.6% after two weeks (during the acute stress time) and 73.8% after four weeks (recovery time). On the forehead, the sebum concentration decreased by 56.4% after two weeks and 80.1% after four weeks (Figure 10).

#### 3.2.3. Skin Exfoliation

**Figure 11:**
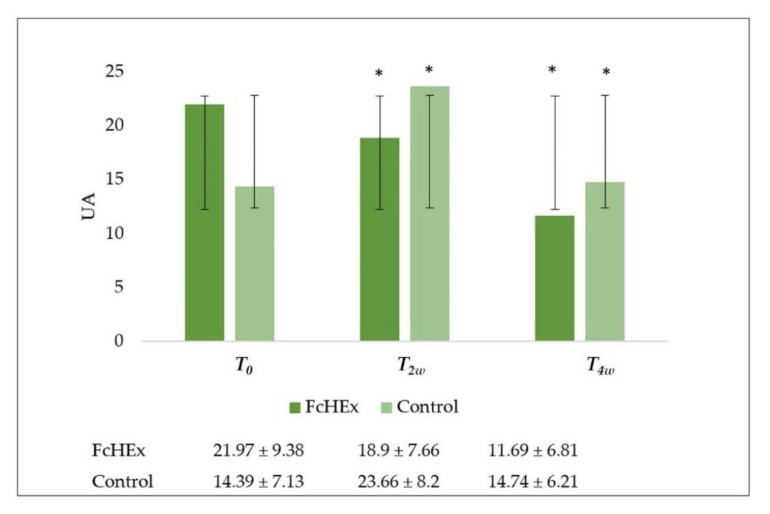
Skin exfoliation evaluation. The corneum exfoliation trend value at time *T_0_* (before the application), *T_2w_* (after two weeks—acute stress), and *T_4w_* (after four weeks—recovery effect). All the measurements for *T_2w_* vs. *T_0_* and *T_4w_* vs. *T_0_* for the FcHEx and the control were statistically significant. The asterisks indicate statistically significant values (* *p*-value was between 0.01 and 0.05).

The skin exfoliation was evaluated using a Corneofix^®^ F 20. After two and four weeks of use, during the acute stress and recovery times, a significant decrease in skin renewal in the volunteers treated with cream containing the FcHEx was recorded (*T_2w_* = −13%, *T_4w_* = −45.7%), and an increase was shown in the volunteers treated with the placebo (*T_2w_* = +84.3%, *T_4w_* = +9.7%) (Figure 11).

#### 3.2.4. Skin Lightness

**Figure 12:**
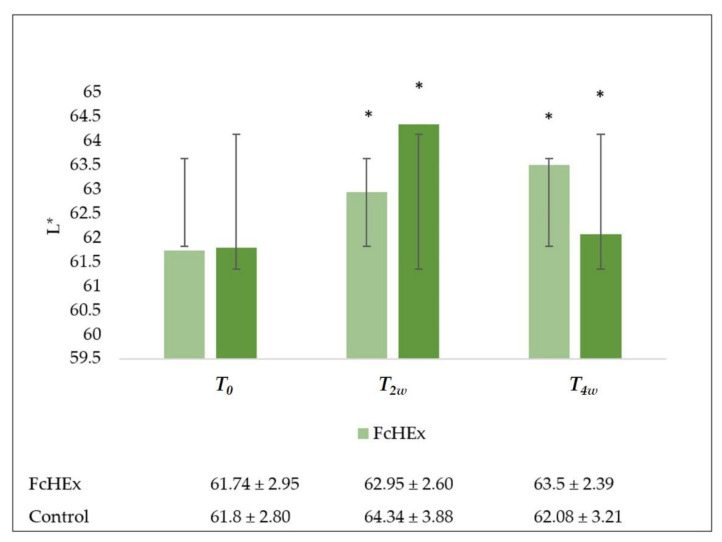
Skin lightness evaluation. Skin lightness trend values at time *T_0_* (before the application), *T_2w_* (after two weeks—acute stress), *T_4w_* (after four weeks—recovery effect). All the measures *T_2W_* vs. *T_0_* and *T_4w_* vs. *T_0_* for the FcHEx and the control were statistically significant. The asterisks indicate statistically significant values (* *p*-value was between 0.01 and 0.05).

The skin lightness was evaluated using a Colorimeter CL 400. The L* value of the volunteers increased in both those who used the cream containing the FcHEx (*T_2w_* = 1.9%, *T_4w_* = 2.7%) and in the placebo users (*T_2w_* = 4.3%, *T_4w_* = 0.8%), but the volunteers that used the placebo showed paler faces than the group used the active cream (Figure 12).

#### 3.2.5. Skin pH

**Figure 13:**
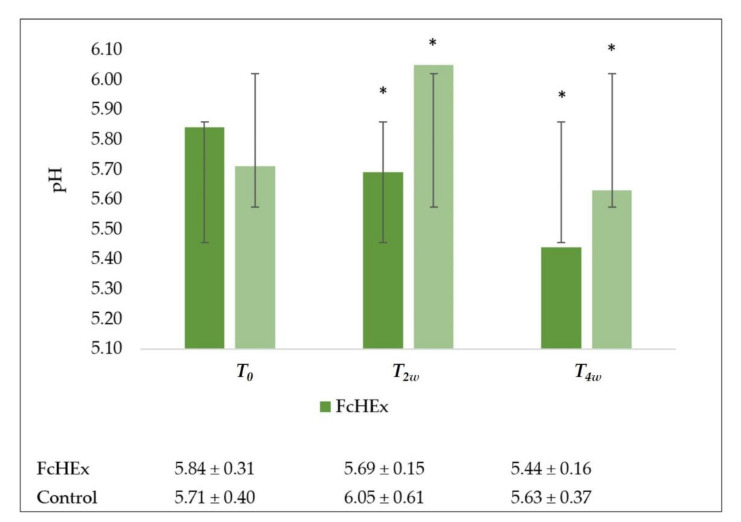
Skin pH evaluation. The skin pH trend values at time *T*_0_ (time of application), *T_2w_* (after two weeks—acute stress), and *T_4w_* (after four weeks—recovery effect). All the measures for *T_2w_* vs. *T_0_* and *T_4w_* vs. *T*_0_ for the FcHEx and the control were statistically significant. The asterisks indicate statistically significant values (* *p*-value was between 0.01 and 0.05).

A skin pH-meter measured the pH on the skin surface. The cream containing the FcHEx maintained the volunteers’ face pH in a normal range (5.5–6) during the acute stress time. In contrast, the placebo increased the pH value during the acute stress time (at *T_2w_* pH = 6.05; Figure 13).

## 4. Discussion

Psychological stress is the process by which the environmental requirements exceed a person’s capacity to adapt, leading to emotional, behavioral, and physiological changes [6]. Chronic stress-induced augmentation in the release of glucocorticoids, epinephrine, and norepinephrine can accelerate aging [25], bacterial colonization, and human peptide protection [26,27]. Stressful conditions, such as those of a chronically ill patient undergoing treatment, might decrease the leukocyte telomere length and telomerase activity, accelerating cellular senescence and decreasing life expectancy [28]. These changes depend on β2-adrenoceptor activation by catecholamines, causing DNA damage and p53 suppression [29]. Oxidation and inflammation processes enhance neutrophil elastase and matrix metalloproteinase-8 (MMP-8) production, which leads to collagen and elastic fiber degradation and wrinkle formation [13]. Research on new cosmetic materials to relieve psychological stress diseases of the skin has been actively pursued. Natural ingredients are generally safer to use than synthetic materials [30]. In this study, a hydrosoluble extract of *Ficus carica* cell suspension cultures (FcHEx), a known source of ficin and phenolic compounds [31], was characterized for its capacity to protect skin cells against the oxidative damage caused by psychological stress. In vitro and in vivo experiments were performed to demonstrate the positive action of the FcHEx that can revert oxidative damage on lipids and proteins due to epinephrine’s stress-induced effect. Skin cells and neurons share a common embryonic origin, as both express proteins that are involved in conserved signaling pathways. Psychological stress triggers the autonomic nervous system to release catecholamines, such as epinephrine and norepinephrine, which can significantly affect many cell types’ metabolisms and functions, including those of the skin [9]. Epinephrine interacts directly with neutrophils, reducing these cell responses to various cAMP-mediated proinflammatory stimuli [32]. The activation of the epinephrine downstream signals was measured in keratinocytes cells to investigate the capacity of the FcHEx to affect the epinephrine signal cascade in skin cells. In keratinocytes, the inflammatory process was induced via epinephrine administration, and the FcHEx significantly reduced the epinephrine stimulation. The cAMP-mediated inflammatory cytokine gene expression was analyzed, and the FcHEx administration reduced the interleukin 6 production by 50%. Moreover, the lipid peroxides production in the keratinocytes was used to test the antioxidant properties of the FcHEx, which showed a significant antioxidant property, decreasing ROS production by 25%. The lipofuscin test confirmed these data as the treatment with both the FcHEx doses entirely abolished the production of the lipofuscin that was mediated by epinephrine. The inhibition of the carbonylated protein concentration by 50% in the keratinocytes showed that the antioxidant properties of the FeHEx were also linked to an inhibition of protein oxidation. Experiments conducted in skin explants confirmed the protective effect of the FcHEx against protein carbonylation, suggesting that the antioxidant action of the FcHEx was mainly linked to an inhibition of protein oxidation, which is the main threat to skin functionality and integrity [33]. These results encouraged us to study the potential role of the FcHEx in the development of the skin hydrolipidic film, which is able to moisturize and stop excessive water loss of the skin [34] and protect the epidermal lipid barrier from microbe attacks and physical injuries, supporting skin integrity. HaCaT cells were treated with the FcHEx to verify its action on the gene expression of GBA, an enzyme that is able to catalyze ceramide production [35]. The results showed that the GBA expression increased in a dose-dependent manner (50% and 75% at the concentrations of 0.002% and 0.006%, respectively). Furthermore, the content of ceramides by Nile red staining in cells stressed with epinephrine and treated with the FcHEx was measured to confirm the gene expression results.

The biological activity of the FcHEx was confirmed via a clinical evaluation on a group of 40 human volunteers, aged between 20 and 27 years, going through a period of psychological stress. For 28 days, twice daily, the volunteers were asked to apply either a cream containing the FcHEx at the concentration of 0.5% or a placebo cream without the ingredient on the whole face. Parameters such as TEWL, sebum flow, skin exfoliation, and lightness, were evaluated. The measurements were carried out during a high-stress condition, namely, a mid-year examination, and after two weeks from the high-stress condition (recovery time). The TEWL value in the volunteers treated with placebo was higher (48.8%) than the volunteers treated with the cream containing the FcHEx (active cream −0.2%), indicating that the extract restored the regular epidermal permeability barrier homeostasis, which was perturbed by the psychological stress. Indeed, the loss of transepidermal water is an indicator of skin barrier integrity since there is a water concentration gradient inside the stratum corneum, which is continuously spread from the body to the skin’s surface and the environment. Thus, low TEWL values are representative of an unaltered skin barrier [36]. Generally, during a student’s psychological stress period due to an approaching examination, acne-related symptoms increase [37], and cellular turnover accelerates, depending on the stress levels [38]. The cream containing the *Ficus carica* extract decreased sebum production and inhibited exfoliation in the clinical tests. Desquamation involves the degradation of corneodesmosomes by enzymes that depend on water and pH for their activity. In the skin, the exfoliation is highest at neutral-to-low alkaline pH levels and decreases at acidic pH levels [39]. The active cream normalized and maintained all volunteers’ face pH in a normal range during the acute stress, which usually rises in stress conditions. A neutral pH impedes the secretion of polar lipids (glucosylceramides) into mature lamellar bilayers (ceramides), enhancing proteolytic activities and increasing corneodesmolysis aberrations in corneocyte cohesion [40]. Moreover, the skin pH influences the peroxidase activity of the ficin, which is one of the components of the FcHEx that is responsible for the antioxidant activity of the extract [41]. The ficin peroxidase activity was around 60% or higher at a pH of 4.5–5.0, the pH of normal skin [42]. Finally, whether the skin turned pale was evaluated to test the cream’s effect on the sympathetic axis (adrenaline and noradrenaline) activated by the acute stress [38]. A small increase in L* was observed in volunteers to whom the cream was applied, suggesting a good efficacy of the extract to prevent the facial skin turning to a pale color due to psychological stress.

## 5. Conclusions

In vitro and in vivo tests demonstrated that the extract from *Ficus carica* cell cultures alleviated skin damage caused by psychological stress. The FcHEx worked as an anti-stress ingredient that alleviated the negative consequences of stress hormone activity on the skin, such as inflammation, oxidation, alteration of the skin barrier, and skin turning to a pale color. All the data support the conclusion that the *Ficus carica* cell culture extract has good potential to be employed in skincare products, particularly those formulated for dry and stressed skins.

## Data Availability

Data is contained within the article or supplementary material.

## References

[1] Dini I., Laneri S. (2019). Nutricosmetics: A brief overview. Phytother. Res..

[2] Laneri S., Di Lorenzo R.M., Bernardi A., Sacchi A., Dini I. (2020). Aloe barbadensis: A Plant of Nutricosmetic Interest. Nat. Prod. Comm..

[3] Laneri S., Di Lorenzo R., Sacchi A., Dini I. (2019). Dosage of bioactive molecules in the nutricosmeceutical Helix aspersa muller mucus and formulation of new cosmetic cream with moisturizing effect. Nat. Prod. Comm..

[4] Wild C.P. (2005). Complementing the genome with an “exposome”: The outstanding challenge of environmental exposure measurement in molecular epidemiology. Cancer Epidemiol. Biomark. Prev..

[5] Krutmann J., Bouloc A., Sore G., Bernard B.A., Passeron T. (2017). The skin aging exposome. Dermatol. Sci. J..

[6] Vileikyte L. (2007). Stress and wound healing. Clin. Dermatol..

[7] Papadimitriou A., Priftis K.N. (2009). Regulation of the hypothalamic-pituitary-adrenal axis. Neuroimmunomodulation.

[8] Cohen S., Janicki-Deverts D., Miller G.E. (2007). Psychological stress and disease. JAMA.

[9] Chen Y., Lyga J. (2014). Brain-skin connection: Stress, inflammation and skin aging. Inflamm. Allergy Drug Targets.

[10] Mammone T., Marenus K., Maes D., Lockshin R.A. (1998). The induction of terminal differentiation markers by the cAMP pathway in human HaCaT keratinocytes. Skin Pharmacol. Physiol..

[11] Grando S.A., Pittelkow M.R., Schallreuter K.U. (2006). Adrenergic and cholinergic control in the biology of epidermis: Physiological and clinical significance. J. Investig. Dermatol..

[12] Choi E.H., Brown B.E., Crumrine D., Chang S., Man M.Q., Elias P.M., Feingold K.R. (2005). Mechanisms by which psychologic stress alters cutaneous permeability barrier homeostasis and stratum corneum integrity. J. Investig. Dermatol..

[13] Romana-Souza B., Santos Lima-Cezar G., Monte-Alto-Costa A. (2015). Psychological stress-induced catecholamines accelerates cutaneous aging in mice. Mech. Ageing Dev..

[14] Santacruz-Perez C., Paulo Newton Tonolli P., Ravagnani F.G., Baptista F.S., Saha S., Mondal S. (2018). Photochemistry of Lipofuscin and the Interplay of UVA and Visible Light in Skin Photosensitivity, Photochemistry and Photophysics. Fundamentals to Applications.

[15] Laneri S., Dini I., Tito A., Di Lorenzo R., Bimonte M., Tortora A., Zappelli C., Angelillo M., Bernardi A., Sacchi A. (2021). Plant cell culture extract of Cirsium eriophorum with skin pore refiner activity by modulating sebum production and inflammatory response. Phytother. Res..

[16] Apone F., Tito A., Arciello S., Carotenuto G., Colucci M.G. (2020). Plant tissue cultures as sources of ingredients for skin care applications. Ann. Plant Rev..

[17] Carlson R.V., Boyd K.M., Webb D.J. (2004). The revision of the Declaration of Helsinki: Past, present and future. Br. J. Clin. Pharmacol..

[18] Renner G., Audebert F., Burfeindt J., Calvet B., Caratas-Perifan M., Leal M.E., Gorni R., Long A., Meredith E., O’Sullivan Ú. (2017). Cosmetics Europe guidelines on the management of undesirable effects and reporting of serious undesirable effects from cosmetics in the European Union. Cosmetics.

[19] Tsalokostas G. (2009). Using Tissue Culture as an Alternative Source of Polyphenols Produced by *Ficus carica* L.. Ph.D. Thesis.

[20] Gagaoua M., Boucherba N., Bouanane-Darenfed A., Ziane F., Nait-Rabah S., Hafid K., Boudechicha H.-R. (2014). Three-phase partitioning as an efficient method for the purification and recovery of ficin from Mediterranean fig (*Ficus carica* L.) latex. Sep. Purif. Technol..

[21] Nassar A.H., Newbury H.J. (1987). Ficin production by callus cultures of *Ficus carica*. Plant Phys. J..

[22] Cuesta A.M., Albiñana V., Gallardo-Vara E., Recio-Poveda L., de Rojas P.I., de Las-Heras K.V.G., Aguirre D.T., Botella L.M. (2019). The b_2-adrenergic receptor antagonist ICI-118,551 blocks the constitutively activated HIF signaling in hemangioblastomas from von Hippel-Lindau disease. Sci. Rep..

[23] Zumwalt J.W., Thunstrom B.J., Spangelo B.L. (1999). Interleukin-1beta and catecholamines synergistically stimulate interleukin-6 release from rat C6 glioma cells in vitro: A potential role for lysophosphatidylcholine. Endocrinology.

[24] Wortsman J., Frank S., Cryer P.E. (1984). Adre-nomedullary response to maximal stress in humans. Am. J. Med..

[25] Dunn J.H., Koo J. (2013). Psychological Stress and skin aging: A review of possible mechanisms and potential therapies. Dermat. Online J..

[26] Scudiero O., Brancaccio M., Mennitti C., Laneri S., Lombardo B., De Biasi M.G., De Gregorio E., Pagliuca C., Colicchio R., Salvatore P. (2020). Human Defensins: A Novel Approach in the Fight against Skin Colonizing Staphylococcus aureus. Antibiotics.

[27] Pero R., Angrisano T., Brancaccio M., Falanga A., Lombardi L., Natale F., Laneri S., Lombardo B., Galdiero S., Scudiero O. (2019). Beta-defensins and analogs in Helicobacter pylori infections: mRNA expression levels, DNA methylation, and antibacterial activity. PLoS ONE.

[28] Damjanovic A.K., Yang Y., Glaser R., Kiecolt-Glaser J.K., Nguyen H., Laskowski B., Beversdorf D.Q., Zou Y., Weng N.P. (2007). Accelerated telomere erosion is associated with a declining immune function of caregivers of Alzheimer’s disease patients. Immun. J..

[29] Hara M.R., Kovacs J.J., Whalen E.J., Rajagopal S., Strachan R.T., Grant W., Towers A.J., Williams B., Lam C.M., Xiao K. (2011). A stress response pathway regulates DNA damage through beta2-adrenoreceptors and beta-arrestin-1. Nature.

[30] Minich D.M., Bland J.S. (2007). A review of the clinical efficacy and safety of cruciferous vegetable phytochemicals. Nutr. Rev..

[31] Cormier F., Charest C., Dufresne C. (1989). Partial purification and properties of proteases from fig (*Ficus carica*) callus cultures. Biotechnol. Lett..

[32] Moore A.R., Willoughby D. (1995). A The role of cAMP regulation in controlling inflammation. Clin. Exp. Immunol..

[33] Rinnerthaler M., Bischof J., Streubel M.K., Trost A., Richter K. (2015). Oxidative Stress in Aging Human Skin. Biomolecules.

[34] Proksch E., Brandner J.M., Jensen J.M. (2008). The skin: An indispensable barrier. Exp. Dermatol..

[35] Feingold K.R. (2007). The role of epidermal lipids in cutaneous permeability barrier homeostasis. J. Lipid Res..

[36] Garg A., Chren M.M., Sands L.P., Matsui M.S., Marenus K.D., Feingold K.R., Elias P.M. (2001). Psychological stress perturbs epidermal permeability barrier homeostasis: Implications for the pathogenesis of stress-associated skin disorders. Arch Dermatol..

[37] Chiu A., Chon S.Y., Kimball A.B. (2003). The response of skin disease to stress: Changes in acne severity vulgaris as affected by examination stress. Arch Dermatol..

[38] Peters E.M. (2016). Stressed skin a molecular psychosomatic update on stress-causes and effects in dermatologic diseases. J. Dtsch. Dermatol. Ges..

[39] Loden M., Loden M., Mailbach H. (2006). Role of topical emollients and moisturizers in the treatment of dry skin barrier disorders. Dry Skin and Moisturizers: Chemistry and Function.

[40] Fluhr J.W., Elias P.M. (2002). Stratum corneum pH: Formation and function of the acid mantle. Exog. Dermatol..

[41] Cho U.M., Choi D.H., Yoo D.S. (2019). Inhibitory Effect of Ficin Derived from Fig Latex on Inflammation and Melanin Production in Skin Cells. Biotechnol. Bioproc. E.

[42] Devaraj K.B., Kumar P.R., Prakash V. (2008). Purification, characterization, and solvent-induced thermal stabilization of ficin from Ficus carica. J. Agric. Food Chem..

